# Effect change of obesity on diabetes depending on measurement: self-reported body mass index from 2012 Community Health Survey vs. directly measured from the Korea National Health and Nutrition Examination Survey

**DOI:** 10.4178/epih/e2015001

**Published:** 2015-01-06

**Authors:** Kyuhyun Yoon, Kyungduk Min, Heeran Chun, Soong-Nang Jang, Sung-il Cho

**Affiliations:** 1 Department of Epidemiology Graduate School of Public Health and Institute of Health and Environment, Seoul National University, Seoul, Korea; 2 School of Health Science, Jungwon University, Goesan, Korea; 3 Nursing Science Research Institute and College of Nursing, Chung-Ang University, Seoul, Korea

**Keywords:** Effect change, Self-reporting data, Obesity, Diabetes mellitus

## Abstract

**OBJECTIVES:**

Obesity is a well-recognized risk factor for type 2 diabetes mellitus (DM) among young and middle-aged adults in South Korea. To elaborate on the association between obesity and DM, subjective data from self-reporting survey or objective data from health examination is generally used. This study was conducted to validate the change of association from using these different measurements.

**METHODS:**

Community Health Survey data and Korea National Health and Nutrition Examination Survey data, as subjective and objective data respectively, were used. Population, resident in Seoul and over 45 aged, were selected for the study and the association between obesity and DM were defined by using multivariate logistic regression model.

**RESULTS:**

In subjective data, DM prevalence was 12.4% (male, 14.7; female, 10.6) and obesity prevalence was 26.0% (male, 29.2; female, 23.4). Whereas, in objective data, DM prevalence was 15.0% (male, 17.8; female, 12.9), and obese population was 32.4% (male, 34.4; female, 30.8). Based on the effect of obesity on DM prevalence from each data, using objective data increased the impact of obesity. Difference of relative risk of obesity between from subjective data and from objective was bigger in female than male and statistically significant.

**CONCLUSIONS:**

The differences of association pattern between subjective and objective data were found, due to higher obesity prevalence in objective data, and discrepancies of socio-economic status. These discrepancies could be inevitable Therefore we have to face them proactively, and understand the different aspect of various variables from different measurement.

## INTRODUCTION

While studies of obesity diagnosed from body mass index (BMI) have been conducted, self-reported anthropometric information regarding the diagnosis of obesity is subject to systematic errors [1-3], and particularly to discrepancies regarding the characteristics of participants. Nevertheless, self-reported information obtained through questionnaire or interview has the advantages of low cost and higher availability; moreover, these surveys are easy to administer and are a good method for studying large numbers of individuals [4].

It is important to assess the prevalence of obesity in various socio-demographic groups. Health policies, programs, and interventions can be more effective if they are targeted at high-risk populations and adopt a practical approach. In South Korea, the leading representative national health survey is the Korea National Health and Nutrition Examination Survey (KNHANES). It consists of an interview for demographic information, including socioeconomic status and health behavior, a health examination including a laboratory test, and a dietary assessment. In contrast, at the regional municipality level, Community Health Surveys (CHS) comprise interviews conducted with computer-assisted personal interviewing (CAPI), and thus all information from CHS is self-reported. Consequently, the prevalence of obesity from each survey might be different.

It is therefore necessary to determine whether different measurements, such as self-reporting and direct measurement, would give rise to different information or values and whether a difference in association among relevant factors would consequently occur. Answering these questions would address common misgivings about the quality of self-reported anthropometric information and might lead to suggestions for how best the results from studies using self-reported data can be interpreted and used.

Obesity is a well-recognized risk factor for type 2 diabetes mellitus (DM) among young and middle-aged adults both worldwide [5-8] and in South Korea [9]. DM is one of the diseases most directly and strongly associated with obesity. It is predicted that there may be a difference in this association between different tools for obesity measurement. This study was conducted to validate the difference in association arising from using different measurements: objective data on the difference in the relationship of obesity with DM prevalence measured by diagnosis of obesity and DM during an actual health examination; and subjective data from respondent’s self-reporting.

## MATERIALS AND METHODS

### Data and study population

Objective data was obtained from the KNHANES conducted in 2012, and the subjective data was obtained from the Korean CHS. The CHS was a cross-sectional interview survey conducted by the Korea Centers for Disease Control and Prevention (KCDC) and each municipality. The questionnaire consisted primarily of questions about personal health behaviors regarding leading causes of health problems in Korean communities as well as health status and disease condition [10].

The target age of this study was 45 years and over, since chronic diseases such as hypertension and DM are relatively rare in younger generations. Only people living in Seoul, South Korea were selected in order to minimize regional effects while maximizing the total number of data. Some data were excluded due to missing values in education level, marital status, self-rated health status, employment status, income, BMI, blood pressure, smoking, and DM morbidity. With these criteria, a total of 587 objective data and 10,833 subjective data were obtained from 8,058 KNHANES and 228,921 CHS, respectively.

### Definition of variables

As explanatory variables, we selected education level, living status, self-rated health, employment status, equalized income, smoking, obesity, and hypertension.

Education level was categorized by the highest school level completed. Living status was defined by whether subjects lived with their spouse at the time, regardless of marital status. Self-rated health status was measured according to a five-point scale: very good, good, moderate, poor, and very poor. We re-categorized this scale into a three-point scale in order to prevent a small sample size in each category. The employment variable was determined by working status, with the employed group including self-employed, employed with salary, and unpaid family workers. The revised household income was calculated from the total household income divided by family size. Smoking status was divided into three groups: current smokers, past smokers, and non-smokers. Non-smokers indicate people who have never experienced smoking or have smoked less than five packs in their lifetime.

All of the above demographic and socioeconomic variables were obtained by interviewing; however, for obesity, DM and hypertension, the definition was different between subjective and objective data. In the subjective data, reported height and weight were used for obesity, and diagnosis experience was used to measure DM and hypertension prevalence. On the other hand, in the objective data, measured height/weight and blood pressure were used for BMI calculation and hypertension, respectively, and the prevalence of DM was measured by laboratory test. Obesity was defined as a BMI ≥25.0 kg/m^2^ according to the standard of World Health Organization/Western Pacific Regional Office [11]. DM was classified as a fasting (8 hours or more) blood glucose level of ≥126 mg/dL or as taking medication for DM. Hypertension was defined as a systolic blood pressure ≥140 mmHg or a diastolic pressure ≥90 mmHg or as taking medication for hypertension.

### Statistical analysis

Frequency analysis and logistic regression models were mainly used in this study.

Frequency analysis was used in order to understand the demographic characteristics of the data and their association with DM prevalence. The associations were examined by chi-square test using univariate analysis. Logistic regression analysis was then conducted using multivariate analysis. In both frequency and logistic analyses, we separated the male and female populations because female’s economic activity rates are generally lower than male’s and because risky health behaviors such as smoking and drinking are underreported among female. To avoid multicollinearity in the regression model, the marital status and employment status variables were excluded due to strong correlations with age and income, respectively. The smoking variable was also excluded in the female model since the results of the frequency analysis indicated a severely unbalanced proportion. SAS version 9.3 (SAS Institute Inc., Cary, NC, USA) was used for all statistical analysis in this study.

## RESULTS

Table 1 shows the demographic and socioeconomic characteristics from the 2012 Seoul CHS data and their association with self-reported DM prevalence. 12.4% of participants had experienced a DM diagnosis, and the reported obesity prevalence was 26.0%. Both DM and obesity prevalence were higher in males than in females. The association between self-reported DM prevalence and levels of each variable was statistically significant except for living status among male and smoking status among female.

Table 2 shows the demographic and socioeconomic characteristics of the 2012 Seoul KNHANES data and their association with measured DM prevalence. Among the participants in the KNHANES, 15% have DM, and 32.4% show obesity. Both DM prevalence and obesity were higher in the male population than in females. The variables which had a significant association with measured DM prevalence were age, education, income, smoking, obesity, and hypertension in the whole population; age, employment, and smoking in the male population; and age, education, living status, income, obesity, and hypertension in females.

In a multivariate logistic regression model for DM prevalence (Table 3), male show higher odds than female (odds ratio [OR], 1.46; 95% confidence interval [CI], 1.21 to 1.76) only in subjective data. All subjects aged over 55 years show higher odds than the reference group (subjects aged 45 to 54 years) in subjective data, and a similar association was noted in the objective data. Though the association in the 55 to 64 years age group appeared not to be statistically significant, it was marginally significant (OR, 2.05; 95% CI, 0.98 to 4.28). Education level was not statistically significant, with only the middle school group showing higher odds than university graduates in objective data (OR, 2.32; 95% CI, 1.00 to 5.40). Groups with lower self-rated health status showed significantly higher odds than the higher self-rated health group, and this tendency was found only in subjective data, whereas the association between income and DM was shown in the objective data. A significant association of obesity and smoking behavior with DM was noted in both sets of data, but the association of hypertension was shown only in subjective data.

In the multivariate logistic regression model of the male population (Table 4), only age difference and smoking behavior showed significant associations with DM in both sets of data. No significant association was found with education level. On the other hand, the association of self-rated health, obesity, and hypertension were shown only in the subjective data. In the female population (Table 4), the association of income level, obesity, and hypertension showed significance in both sets of data, but when it came to income level, only the second quartile group had the association in subjective data. In contrast, age, education level, and self-rated health showed a significant association only in subjective data.

For both male and female, self-rated health was significant only in the subjective data. When we contrasted results from subjective and objective data, for male, the ORs of age and smoking in the objective data were considerably higher than in the subjective data. In female, the ORs of income, obesity, and hypertension in the objective data were statistically significant and were higher than in the subjective data.

## DISCUSSION

According to subjective data obtained from residents aged 45 years and older living in Seoul, DM prevalence was 12.4% (male, 14.7; female, 10.6). The proportion of the population who were obese (BMI≥25 kg/m^2^) was 26.0% (male, 29.2; female, 23.4). In contrast, according to objective data from the KNHANES from residents aged 45 years and older and living in Seoul, DM prevalence was 15.0% (male 17.8, female 12.9), and the prevalence of obesity (BMI≥25 kg/m^2^) was 32.4% (male, 34.4; female, 30.8). The discrepancy in DM prevalence between the two sets of data was just 2.6%, but that of the obesity prevalence was 6.4%, a difference more than twice that of DM.

Based on the degree of the relationship of obesity with DM prevalence observed in each set of data, we can infer that using objective data increased the observed impact of obesity. The difference in the relative risk of obesity between subjective and objective data was greater in female than in male and all of the relative risk was statistically significant. This appearance is most likely due to the greater prevalence of obesity in the objective data. It should be also noted that other socioeconomic information was collected from the questionnaires in both sets of data. Therefore, some socioeconomic factors in reporting error and in DM prevalence are entangled in the multi directional interaction among them.

In this study, which compares subjective data with objective data, measurement discrepancies in both obesity, the covariate, and DM, the outcome variable, was shown. These discrepancies should result in variation in the association between the survey methods. However, it is very difficult to determine which method is more accurate, since every survey procedure has problems leading to systematic errors.

As for the evaluation of obesity using BMI calculated from height and weight, self-reported and measured values for height and weight are strongly correlated; thus, many studies assume that self-reported data are valid [12-14]. However, researchers have expressed concerns regarding discrepancies and systematic errors in self-reported values, as these are unreliable in population subgroups with a high prevalence of obesity (e.g., overweight female and middle-aged and elderly individuals) [1,2, 15-18]. Being overweight is also a predictor of errors in reporting height measurements [3,19]. In addition to intentional reporting errors, many people are unaware of anthropometric changes such as atrophy after middle age, particularly female and older adults [20]. In fact, systematic reporting errors can result from both intended and unintended misreporting, affecting data reliability and, in turn, introducing bias.

Furthermore, measurements of height and weight have normal circadian variation [21,22]. The accuracy of measurement instrument, and competence of personnel are critical in spite of regular quality control [23]. Selection bias is a well-known problem in population studies. Participants in surveys are likely to be different from those who decline to participate. However, it is not understood how self-selection affects the information. Individuals who are more obese and at greater risk of health problems tend to refuse participation [24]. The time and effort required for measuring and examination may also influence selection bias. In this way, epidemiological surveys engaging with obesity and other features related to the social desirability of health outcomes may be lacking an important portion of the normal distribution of the population.

Accordingly, the measuring tool difference must be highlighted before the results of such surveys are compared and interpreted for any political decision. This point is very important for decision makers to assign a priority to public health policy, practice, or intervention.

Because self-perceptions and social implications of obesity are in the background of self-reports, understanding the potential influences of personal characteristics such as sex, age, ethnicity, and sociocultural and socioeconomic status are important for designing studies and for interpreting data obtained from self-reported information [25,26]. To determine which factors influence certain health outcomes, researchers collect measurements of related, interactive, and potential causes. Some factors present in the survey process need to be considered when researchers use data from a survey and interpret results from each set of data. These include sampling frames, data collection modes (CAPI, computer-assisted self-interviewing, paper-assisted personal interviewing, etc.), and the non-response rate [27].

The goal of CHS is to produce health indicators that are comparable among municipalities, for monitoring community health status. The community level of health indicators indicated by the monitoring of results from CHS in each municipality is the principle evidence used to develop community health plans, intervention planning, outcome assessments and effect evaluations. This goal seems to have been achieved successfully, based on a review of annual community health figures in each municipality since the year 2008 [28]. However, many researchers hesitate to use CHS data due to its data collection method, that is, interviewing with a questionnaire. The KNHANES is the leading nationally representative health survey that provides directly measured information on community-dwelling people through laboratory or physical exams. Generally, directly measured information seems more accurate than reported information because of reduced reporting error, and thus many Korean researchers use data from the KNHANES rather than from CHS. Many previous studies, however, have also provided insights into significant variability in biomarkers from laboratory and physical health exams [22,29-31]. Moreover, the self-reporting method is still used in population-based studies for its low cost and relative ease in practice [4,32-34].

It is worth asking whether the lack of information from particular populations due to differences in survey design and execution is reversible. We should take into account that biased data can be adjusted to a comparably accurate level through sex and age standardization. Now, we need a debate on how we understand and improve measurement bias arising from regional differences in demographic structures. We should do all we can to diminish bias in the design and administration of surveys and be consistent in reporting the level of accuracy of estimation and potential for making prediction from each survey. There is no perfect survey, since measurement error is an indispensable part of surveys. We have to face the discrepancy among different measuring tools proactively, and develop a comprehensive understanding of the different factors involved in the variables from different measurements. Therefore, we call for widespread use of the thus obtained information and strong consideration of the effect of measurement method on results.

## Figures and Tables

**Table 1.** Characteristics of participants and diabetes mellitus (DM) prevalence in the 2012 Seoul metropolitan Community Health Survey

Variables		All		Male		Female	
		n (%)	DM	n (%)	DM	n (%)	DM
			n (%)		n (%)		n (%)
N		10,833 (100)	1,346 (12.4)	4,810 (44.4)	709 (14.7)	6,023 (55.6)	637 (10.6)
Sex	Male	4,810 (44.4)	709 (14.7)^***^				
	Female	6,023 (55.6)	637 (10.6)				
Age	45-54	4,185 (38.6)	214 (5.1)^***^	1,836 (38.2)	133 (7.2)^***^	2,349 (39.0)	81 (3.5)^***^
	55-64	3,458 (31.9)	469 (13.6)	1,496 (31.1)	255 (17.1)	1,962 (32.6)	214 (10.9)
	65-74	2,294 (21.2)	464 (20.2)	1,092 (22.7)	232 (21.3)	1,202 (20.0)	232 (19.3)
	≥ 75	896 (8.3)	199 (22.2)	386 (8.0)	89 (23.1)	510 (8.5)	110 (21.6)
Education	Primary	2,494 (23.0)	486 (19.5)^***^	699 (14.5)	144 (20.6)^***^	1,795 (29.8)	342 (19.1)^***^
	Middle	1,737 (16.0)	239 (13.8)	720 (15.0)	138 (19.2)	1,017 (16.9)	101 (9.9)
	High	3,651 (33.7)	361 (9.9)	1,637 (34.0)	217 (13.3)	2,014 (33.4)	144 (7.2)
	University	2,951 (27.2)	260 (8.8)	1,754 (36.5)	210 (12.0)	1,197 (19.9)	50 (4.2)
Living	With spouse	8,496 (78.4)	1,026 (12.1)^*^	4,255 (88.5)	629 (14.8)	4,241 (70.4)	397 (9.4)^***^
	Without spouse	2,337 (21.6)	320 (13.7)	555 (11.5)	80 (14.4)	1,782 (29.6)	240 (13.5)
Self-rated health	Good	3,794 (35.0)	226 (6.0)^***^	1,938 (40.3)	147 (7.6)^***^	1,856 (30.8)	79 (4.3)^***^
	Moderate	4,689 (43.3)	474 (10.1)	2,014 (41.9)	272 (13.5)	2,675 (44.4)	202 (7.6)
	Poor	2,350 (21.7)	646 (27.5)	858 (17.8)	290 (33.8)	1,492 (24.8)	356 (23.9)
Employment	Employed	5,897 (54.4)	538 (9.1)^***^	3,411 (70.9)	384 (11.3)^***^	2,486 (41.3)	154 (6.2)^***^
	Not employed	4,936 (45.6)	808 (16.4)	1,399 (29.1)	325 (23.2)	3,537 (58.7)	483 (13.7)
Income^1^	Bottom quarter	2,716 (25.1)	483 (17.8)^***^	1,111 (23.1)	224 (20.2)^***^	1,605 (26.7)	259 (16.1)^***^
	2nd	2,701 (24.9)	360 (13.3)	1,193 (24.8)	184 (15.4)	1,508 (25.0)	176 (11.7)
	3rd	2,901 (26.8)	285 (9.8)	1,345 (28.0)	154 (11.5)	1,556 (25.8)	131 (8.4)
	Top quarter	2,515 (23.2)	218 (8.7)	1,161 (24.1)	147 (12.7)	1,354 (22.5)	71 (5.2)
Smoking	Never	6,844 (63.2)	734 (10.7)^***^	1,105 (23.0)	136 (12.3)^***^	5,739 (95.3)	598 (10.4)
	Current	1,764 (16.3)	237 (13.4)	1,600 (33.3)	217 (13.6)	164 (2.7)	20 (12.2)
	Past	6,844 (20.5)	375 (16.9)	2,105 (43.8)	356 (16.9)	120 (2.0)	19 (15.8)
Obesity^2^	Non-obese	8,019 (74.0)	879 (11.0)^***^	3,404 (70.8)	464 (13.6)^***^	4,615 (76.6)	415 (9.0)^***^
	Obese	2,814 (26.0)	467 (16.6)	1,406 (29.2)	245 (17.4)	1,408 (23.4)	222 (15.8)
Hypertension	Yes	3,489 (32.2)	789 (22.6)^***^	1,648 (34.3)	406 (24.6)^***^	1,841 (30.6)	383 (20.8)^***^
	No	7,344 (67.8)	557 (7.6)	3,162 (65.7)	303 (9.6)	4,182 (69.4)	254 (6.1)

All p values was calculated by chi-square tests.

1 Equivalized household income weighted by family number.

2 Based on body mass index (BMI) from reported height and weight (≥ BMI 25).

* p<0.05,

** p<0.01,

*** p<0.001.

**Table 2.** Characteristics of participants and diabetes mellitus (DM) prevalence in the 2012 Seoul Korea National Health and Nutrition Examination Survey

Variables		All		Male		Female	
		n (%)	DM	n (%)	DM	n (%)	DM
			n (%)		n (%)		n (%)
Total		587 (100)	88 (15.0)	253 (43.1)	45 (17.8)	334 (56.9)	43 (12.9)
Sex	Male	253 (43.1)	45 (17.8)				
	Female	334 (56.9)	43 (12.9)				
Age	45-54	191 (32.5)	14 (7.3)^***^	77 (30.4)	6 (7.8)^*^	114 (34.1)	8 (7.0)^*^
	55-64	182 (31.0)	25 (13.7)	74 (29.3)	13 (17.6)	108 (32.3)	12 (11.1)
	65-74	158 (26.9)	37 (23.4)	75 (29.6)	19 (25.3)	83 (24.6)	18 (21.7)
	75-	56 (9.5)	12 (21.4)	27 (10.7)	7 (25.9)	29 (8.7)	5 (17.2)
Education	Primary	152 (25.9)	28 (18.4)^*^	41 (16.2)	7 (17.1)	111 (33.2)	21 (18.9)^*^
	Middle	82 (14.0)	18 (22.0)	31 (12.3)	8 (25.8)	51 (15.3)	10 (19.6)
	High	195 (33.2)	28 (14.4)	91 (36.0)	21 (23.1)	104 (31.1)	7 (6.7)
	University	158 (26.9)	14 (8.9)	90 (35.6)	9 (10.0)	68 (20.4)	5 (7.4)
Living	With spouse	491 (83.7)	68 (13.9)	240 (94.9)	42 (17.5)	251 (75.2)	26 (10.4)^*^
	Without spouse	96 (16.4)	20 (20.8)	13 (5.1)	3 (23.1)	83 (24.9)	17 (20.5)
Self-rated health	Good	176 (30.0)	20 (11.4)	94 (37.2)	14 (14.9)	82 (24.6)	6 (7.3)
	Moderate	303 (51.6)	48 (15.8)	123 (48.6)	25 (20.3)	180 (53.9)	23 (12.8)
	Poor	108 (18.4)	20 (18.5)	36 (14.2)	6 (16.7)	72 (21.6)	14 (19.4)
Employment	Employed	283 (48.2)	34 (12.0)	158 (62.5)	20 (12.7)^**^	125 (37.4)	14 (11.2)
	Not employed	304 (51.8)	54 (17.8)	95 (37.6)	25 (26.3)	209 (62.6)	29 (13.9)
Income^1^	Bottom quarter	148 (25.2)	30 (20.3)^**^	56 (22.1)	13 (23.2)	92 (27.5)	17 (18.5)^**^
	2nd	147 (25.0)	28 (19.1)	70 (27.7)	15 (21.4)	77 (23.1)	13 (16.9)
	3rd	150 (25.6)	21 (14.0)	65 (25.7)	10 (15.4)	85 (25.5)	11 (12.9)
	Top quarter	142 (24.2)	9 (6.3)	62 (24.5)	7 (11.3)	80 (24.0)	2 (2.5)
Smoking	Never	366 (62.4)	41 (11.2)^**^	53 (21.0)	3 (5.7)^*^	313 (93.7)	38 (12.1)
	Current	83 (14.1)	17 (20.5)	70 (27.7)	14 (20.0)	13 (3.9)	3 (23.1)
	Past	138 (23.5)	30 (21.7)	130 (51.4)	28 (21.5)	8 (2.4)	2 (25.0)
Obesity^2^	Non-obese	397 (67.6)	46 (11.6)^***^	166 (65.6)	26 (15.7)	231 (69.2)	20 (8.7)^***^
	Obese	190 (32.4)	42 (22.1)	87 (34.4)	19 (21.8)	103 (30.8)	23 (22.3)
Hypertension	Yes	253 (43.1)	55 (21.7)^***^	118 (46.6)	26 (22.0)	135 (40.4)	29 (21.5)^***^
	No	334 (56.9)	33 (9.9)	135 (53.4)	19 (14.1)	199 (59.6)	14 (7.0)

All p values was calculated by chi-square tests.

1 Equivalized household income weighted by family number.

2 Based on body mass index (BMI) from reported height and weight (≥ BMI 25).

* p<0.05,

** p<0.01,

*** p<0.001.

**Table 3.** Relative risk from multiple logistic regression analysis for diabetes mellitus prevalence

Variables		Subjective	Objective
Sex	Male	1.46 (1.21, 1.76)^*^	0.55 (0.24, 1.26)
	Female	1.00 (reference)	1.00 (reference)
Age	45-54	1.00 (reference)	1.00 (reference)
	55-64	2.19 (1.83, 2.62)^*^	2.05 (0.98, 4.28)
	65-74	2.69 (2.21, 3.28)^*^	3.32 (1.55, 7.09)^*^
	75-	2.50 (1.96, 3.18)^*^	3.21 (1.18, 8.71)^*^
Education	Primary	1.15 (0.94, 1.40)	1.13 (0.50, 2.55)
	Middle	1.05 (0.85, 1.30)	2.32 (1.00, 5.40)^*^
	High	0.98 (0.81, 1.17)	1.15 (0.55, 2.42)
	University	1.00 (reference)	1.00 (reference)
Self-rated health	Good	1.00 (reference)	1.00 (reference)
	Moderate	1.66 (1.40, 1.96)^*^	1.48 (0.81, 2.69)
	Poor	4.37 (3.67, 5.22)^*^	1.37 (0.66, 2.86)
Income^1^	Bottom quarter	0.98 (0.80, 1.20)	2.22 (0.92, 5.33)
	2nd	1.07 (0.88, 1.31)	2.39 (1.02, 5.58)^*^
	3rd	0.95 (0.78, 1.16)	2.41 (1.01, 5.72)^*^
	Top quarter	1.00 (reference)	1.00 (reference)
Smoking	Never	1.00 (reference)	1.00 (reference)
	Current	1.30 (1.05, 1.61)^*^	4.21 (1.69, 10.53)^*^
	Past	1.28 (1.05, 1.57)^*^	3.55 (1.49, 8.50)^*^
Obesity^2^	Non-obese	1.00 (reference)	1.00 (reference)
	Obese	1.40 (1.23, 1.60)^*^	2.08 (1.25, 3.45)^*^
Hypertension	Yes	2.15 (1.89, 2.45)^*^	1.56 (0.92, 2.65)
	No	1.00 (reference)	1.00 (reference)

Values are presented as odds ratio (95% confidence interval).

1 Equivalized household income weighted by family number.

2 Based on body mass index (BMI) from reported height and weight (≥ BMI 25).

* p<0.05

**Table 4.** Relative risk from multiple logistic regression analysis for diabetes mellitus prevalence in sex

Variables		Male		Female	
		Subjective	Objective	Subjective	Objective
Age	45-54	1.00 (reference)	1.00 (reference)	1.00 (reference)	1.00 (reference)
	55-64	2.13 (1.68, 2.71)^*^	3.14 (1.04, 9.52)^*^	2.27 (1.71, 3.00)^*^	1.69 (0.61, 4.68)
	65-74	2.41 (1.85, 3.14)^*^	4.53 (1.46, 14.08)^*^	3.03 (2.23, 4.10)^*^	2.27 (0.81, 6.37)
	75-	2.18 (1.55, 3.08)^*^	5.78 (1.40, 24.04)^*^	2.83 (1.99, 4.03)^*^	1.61 (0.39, 6.71)
Education	Primary	0.95 (0.72, 1.25)	0.95 (0.29, 3.09)	1.43 (1.01, 2.05)^*^	1.01 (0.30, 3.43)
	Middle	1.10 (0.84, 1.44)	3.05 (0.92, 10.14)	1.16 (0.79, 1.69)	1.55 (0.44, 5.40)
	High	0.95 (0.84, 1.44)	1.91 (0.75, 4.91)	1.18 (0.83, 1.67)	0.57 (0.16, 2.09)
	University	1.00 (reference)	1.00 (reference)	1.00 (reference)	1.00 (reference)
Self-rated health	Good	1.00 (reference)	1.00 (reference)	1.00 (reference)	1.00 (reference)
	Moderate	1.74 (1.40, 2.16)^*^	1.3 (0.58, 2.89)	1.54 (1.17, 2.03)^*^	1.58 (0.58, 4.32)
	Poor	4.77 (3.77, 6.05)^*^	0.74 (0.24, 2.32)	3.97 (3.03, 5.21)^*^	1.93 (0.65, 5.71)
Income^1^	Bottm quarter	0.80 (0.60, 1.05)	1.36 (0.42, 4.46)	1.31 (0.96, 1.79)	5.25 (1.07, 25.81)^*^
	2nd	0.86 (0.66, 1.13)	1.43 (0.49, 4.16)	1.46 (1.07, 1.99)^*^	4.91 (1.01, 23.97)^*^
	3rd	0.74 (0.57, 0.96)^*^	1.34 (0.43, 4.13)	1.35 (0.98, 1.85)	5.03 (1.03, 24.50)^*^
	Top quarter	1.00 (reference)	1.00 (reference)	1.00 (reference)	1.00 (reference)
Smoking	Never	1.00 (reference)	1.00 (reference)		
	Current	1.30 (1.02, 1.67)^*^	5.94 (1.46, 24.14)^*^		
	Past	1.30 (1.04, 1.63)^*^	4.74 (1.24, 18.09)^*^		
Obesity^2^	Non-obese	1.00 (reference)	1.00 (reference)	1.00 (reference)	1.00 (reference)
	Obese	1.37 (1.14, 1.65)^*^	1.43 (0.67, 3.06)	1.35 (1.12, 1.63)^*^	2.46 (1.21, 5.00)^*^
Hypertension	Yes	2.10 (1.76, 2.51)^*^	1.23 (0.58, 2.59)	2.18 (1.81, 2.63)^*^	2.22 (1.02, 4.82)^*^
	No	1.00 (reference)	1.00 (reference)	1.00 (reference)	1.00 (reference)

Values are presented as odds ratio (95% confidence interval).

1 Equivalized household income weighted by family number.

2 Based on body mass index (BMI) from reported height and weight (≥ BMI 25).

* p<0.05
